# Extracellular Vesicles in Cervical Cancer and HPV Infection

**DOI:** 10.3390/membranes11060453

**Published:** 2021-06-20

**Authors:** Víctor Acevedo-Sánchez, Ruth M. Rodríguez-Hernández, Sergio R. Aguilar-Ruíz, Honorio Torres-Aguilar, María de los A. Romero-Tlalolini

**Affiliations:** 1 Facultad de Medicina y Cirugía, Universidad Autónoma Benito Juárez de Oaxaca, Ex Hacienda de Aguilera S/N, Calz. San Felipe del Agua, Oaxaca de Juárez 68120, Oaxaca, Mexico; victor_acevedo_schz@hotmail.com (V.A.-S.); monserratrh95@gmail.com (R.M.R.-H.); sar_cinvestav@hotmail.com (S.R.A.-R.); 2 Facultad de Ciencias Químicas, Universidad Autónoma Benito Juárez de Oaxaca, Av. Universidad S/N, Cinco Señores, Oaxaca de Juárez 68120, Oaxaca, Mexico; qbhonorio@hotmail.com; 3 Facultad de Medicina y Cirugía, CONACYT—Universidad Autónoma Benito Juárez de Oaxaca, Ex Hacienda de Aguilera S/N, Calz. San Felipe del Agua, Oaxaca de Juárez 68120, Oaxaca, Mexico

**Keywords:** HPV, exosomes, extracellular vesicles, exosomal content, E6/E7, cancer

## Abstract

Since their description, extracellular vesicles (EVs) have shown growing relevance in cancer progression. These cell structures contain and transfer molecules such as nucleic acids (including DNA and RNA), proteins, and lipids. Despite the rising information about EVs’ relationship with cancer, there is still scarce evidence about their content and function in cervical cancer. Interestingly, the composition and purposes of some cellular molecules and the expression of oncogenic proteins packaged in EVs seem modified in HPV-infected cells; and, although only the E6 oncogenic protein has been detected in exosomes from HPV-positive cells, both E6/E7 oncogenes mRNA has been identified in EVs; however, their role still needs to be clarified. Given that EVs internalizing into adjacent or distant cells could modify their cellular behavior or promote cancer-associated events like apoptosis, proliferation, migration, or angiogenesis in receptor cells, their comprehensive study will reveal EV-associated mechanisms in cervical cancer. This review summarizes the current knowledge in composition and functions of cervical cancer and HPV Infection-derived EVs.

## 1. Introduction

Extracellular vesicles (EVs) have been considered essential for developing several kinds of cancer and other pathologies in the last years. To date, there are thousands of articles evidencing EVs´ involvement in cancer progression [1,2]. However, studying cancer in such a general way is both impossible and inaccurate, so this review focuses on cervical cancer, which is still considered a global public health problem [3].

In 2020, 341,831 women died from cervical cancer all around the world. Among all types of cancer, 3.4% correspond to deaths from cervical cancer worldwide [4]. Cervical cancer is associated with high-risk human papillomavirus (HR-HPV) infection in 99.7% of cases [5]. HR-HPVs are considered the main predisposing factor, but it is not sufficient by itself for cancer evolution [6]. Cancer progression depends, among other factors, on multiple heterotypic cell interactions forming the tumor environment [7]. Thus, in addition to HPV infection, the contribution of several additional factors has been analyzed [6]. Recently, some analyses have evaluated the role of EVs in cancer development since these EVs lead to cellular communication through transferring their content from a donor to a receptor cell and the consequent modification of cellular processes allowing tumor progression [8].

EVs’ existence was suggested in 1946, but was only widely demonstrated in the last four decades. Since their description, EVs have shown increasing relevance in cancer progression [9,10,11]. According to the International Society of Extracellular Vesicles (ISEV) definition, EVs are particles naturally released by cells. These elements are delimited by a lipid bilayer, do not contain a functional nucleus, and cannot replicate [12]. Considering their biogenesis, the main types of EVs include exosomes, microvesicles (MVs), and apoptotic bodies [8,11].

When cells incorporate extracellular milieu content through early endosomes by invagination of the cell plasma membrane, these early endosomes mature into late endosomes by fusion with other vesicles. Then, multivesicular bodies (MVBs) arise by internal budding of the endosomal membrane to develop intraluminal vesicles, the future exosomes. This process is mediated by the Endosomal Sorting Complex Required for Transport (ESCRT) or a ceramide-dependent mechanism. Finally, MVBs fuse with the cell membrane releasing the exosomes into the extracellular space [1,13]. MVs are generated from the direct cytoplasmic membrane budding through cytoskeleton reorganization in an intracellular calcium concentration-dependent process. Increased intracellular calcium changes plasma membrane phospholipids distribution and activates cytosolic proteins involved in cytoskeleton remodeling. Then, the cytoskeleton-bound membrane is disrupted, and contractile, and cleavage proteins such as calpain are recruited, allowing membrane blebs formation and microvesicles release [2].

EVs contain different molecules such as nucleic acids, proteins, lipids, and metabolites. Transferring of EVs’ content promotes reprogramming of recipient cell functions [8,11]. EVs’ interaction with target cells can be through different mechanisms such as membrane fusion, endocytosis, phagocytosis, or cell membrane molecules [8,13].

EVs are enriched in some molecules such as cytoskeletal proteins, class I and II major histocompatibility complex (MHC) proteins, adhesion proteins (tetraspanins, integrins), heat shock proteins, and membrane fusion proteins (Rab, annexins) [8]. Proteins packing and sorting in EVs is a regulated process, where some proteins are involved in their biogenesis and secretion of a particular kind of vesicles; hence, this can be used as a marker to identify the EVs type [14]. Regarding nucleic acids, these EVs contain high molecular weight double-stranded DNA, and in cancer patients, EVs are enriched with tumor DNA [8]. The proposed mechanism for DNA loading in EVs is through micronuclei, which, due to their instability, collapses and exposes their content to the cytoplasm, where DNA could be transported to the MVBs by interaction with CD63 tetraspanin and finally loaded into exosomes [15]. Additionally, EVs are also enriched with several non-coding RNAs, mainly miRNAs. It has been proposed that these miRNAs could be packaged by different mechanisms, such as the neutral sphingomyelinase-dependent (nSMase2) exosomal transfer, sequence motifs recognition in miRNAs by post-translationally modified ribonucleoproteins, and by a pathway dependent on the post-transcriptional modification of the 3′ end of miRNAs [16,17,18].

Despite all the available information on EVs’ involvement in cancer, there are currently less than 50 experimental papers about their content in cervical cancer or the relationship between EVs and HPV-associated cervical cancer [8,19,20,21]. This evidence reveals the EVs’ content from cervical cancer samples and cell lines. The effect of these EVs has been evaluated both in vivo and in vitro. The results open a new and extensive field to understand the disease progression and seek future clarifications to this or other related conditions. Here, we briefly summarize current advances in EVs’ content and their function in HPV infections and cervical cancer. A summary of EVs’ molecules, the kind of vesicle, and origin (cells or samples) are listed in Table 1.

## 2. Protein Cargo in Extracellular Vesicles

One of the main components of EVs is proteins. These elements can be found within the lumen or in their membrane. The first report about protein cargo in EVs related to cervical cancer was made by Khan et al. in 2011. The authors evidenced that survivin, a member of the inhibitor of apoptosis (IAP) family, was released through exosomes by cervical cancer cells (HeLa). Furthermore, according to their role in chemoresistance, survivin significantly increased when a sublethal dose of proton irradiation was administered. This increase was independent of the amount of EVs released since there was no increase in EVs’ secretion [22]. These results might indicate a possible induction of chemoresistance in other receptor cells, but it still needs demonstration. The survivin and other members of the IAP family (XIAP, c-IAP1, and livin) were later identified in EVs from cervical cancer [23]. The authors also demonstrated the influence of HPV in the protein cargo of EVs and the amount of EVs secreted by silencing the E6/E7 HPV18 oncogenes in the HeLa cell line. After silencing, they observed an increase in the EVs released and a decrease in the survivin levels. The XIAP, c-IAP1, and livin proteins in EVs were also increased [23]. This evidence suggests a potential role of EVs in apoptosis and chemoresistance, but EVs’ protein content could also affect several other processes. Some oxidative stress-related proteins have been reported in EVs of cervical cancer, such as the cytochrome P450 family proteins CYP1A1, CYP1B, and CYP2A6, as well as the antioxidant enzyme superoxide dismutase 1 (SOD1). These EVs isolated from the CaSki cell line are taken up by the U1 cell line of macrophages infected with HIV-1 and increase HIV-1 replication through the CYP pathways and oxidative stress by increasing ROS levels and decreasing antioxidant capacity, cell death, and DNA damage in U1 cells [24].

Recently, Liang et al. identified the Wnt-2b protein in exosomes derived from cervical cancer cell lines. These exosomes are taken up by fibroblasts in vitro and in vivo, promoting their activation and conversion to cancer-associated fibroblasts through the Wnt/β-catenin signaling pathway, increasing migration and proliferation of these cells. Wnt-2b is also found at high levels in serum exosomes from cervical cancer patients compared to healthy controls. Uptake of these exosomes by normal fibroblasts increases expression levels of α-SMA, fibroblast activation protein (FAP), and total and non-phosphorylated β-catenin [25].

Other protein cargo could also contribute to metastasis after the internalization of exosomes. The exosomes of cervical cancer taken up by HUVEC cells can alter vascular integrity and facilitate metastasis through the generation of endoplasmic reticulum stress and the decrease in the expression of zonula occludens-1 (ZO-1) and claudin-5 (CLDN5) proteins, both associated with tight junctions. The stress on the endoplasmic reticulum is attributed to the exosome protein content. A group of 44 proteins was identified in exosomes through a mass spectrometry analysis, including CHMP4B, STX-7, and RPL28, considered as potential targets of this effect. Furthermore, injecting these exosomes in mice increases vascular permeability and tumor metastasis in vivo [26].

In other cancers, oncogenic viruses-derived proteins such as Epstein-Barr virus, hepatitis B virus, or hepatitis C virus have been identified in exosomes [27,28,29]. Nevertheless, neither the direct effect of the viral proteins packaging in EVs on recipient cells nor the mechanisms are fully understood [28,29,30]. The research about HPV viral proteins in EVs from cervical cancer is scarce. Honegger et al. evaluated the presence of E6 and E7 HPV18 oncoproteins in EVs isolated from the HeLa cell line. However, these oncoproteins were not identified. Despite this result, they did not rule out their existence in EVs and suggested that using more sensitive techniques might evidence them [23]. Recently Ranjit et al. also evaluated the presence of these oncoproteins in EVs derived from the CaSki cell line, and they identified the E6 oncoprotein; but, the E7 oncoprotein was not identified [24].The presence of the E6/E7 oncoproteins packaged in EVs from different cell lines and samples needs to be evaluated due to their potential effect on keratinocytes and other cell types. The HPVs have been related to cervical cancer and others such as vulva, anus, vagina, penis, and head and neck [31]. The exosomes isolated from the head and neck cancer cell lines UM-SCC-2, UM-SCC-47, and UPCI-SCC-90, contain the E6 and E7 HPV16 oncoproteins, in addition to other proteins such as p16, survivin, Rb, cyclin D1, p53, SHP-2, and the immunoregulatory proteins TGF-β, FasL, OX40, and OX40L. When co-incubated with T lymphocytes, these exosomes suppressed their activation and proliferation and induced apoptosis, but their effect on immature dendritic cells promoted their maturation and did not affect the expression of antigen processing machinery components [32]. An indirect CD8+ T cells cytotoxicity suppression induced by EVs has also been observed when Langerhans cells (LCs) are co-cultured with MVs derived from E7-expressing keratinocytes. When these LCs were activated with LPS, the CD40 and IL-12 expression were reduced; the authors attributed this reduced T cells response due to the modified antigen-presentation properties of LCs [33].

**Figure 1:**
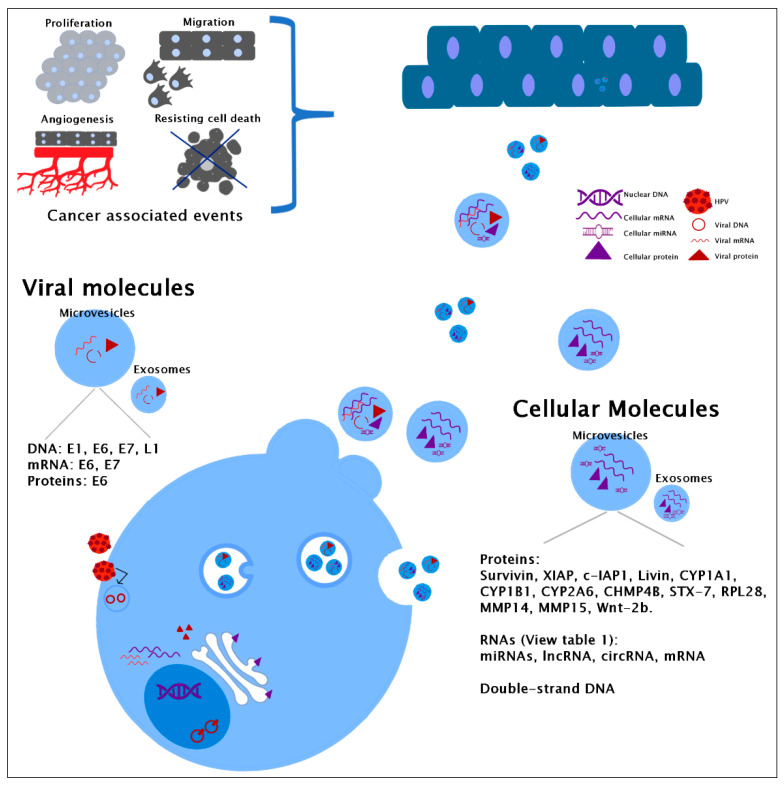
Cellular and viral molecules identified in extracellular vesicles. Only stratified tissue is represented in the figure, but keratinocytes and other cells can also internalize the vesicles.

**Figure 2:**
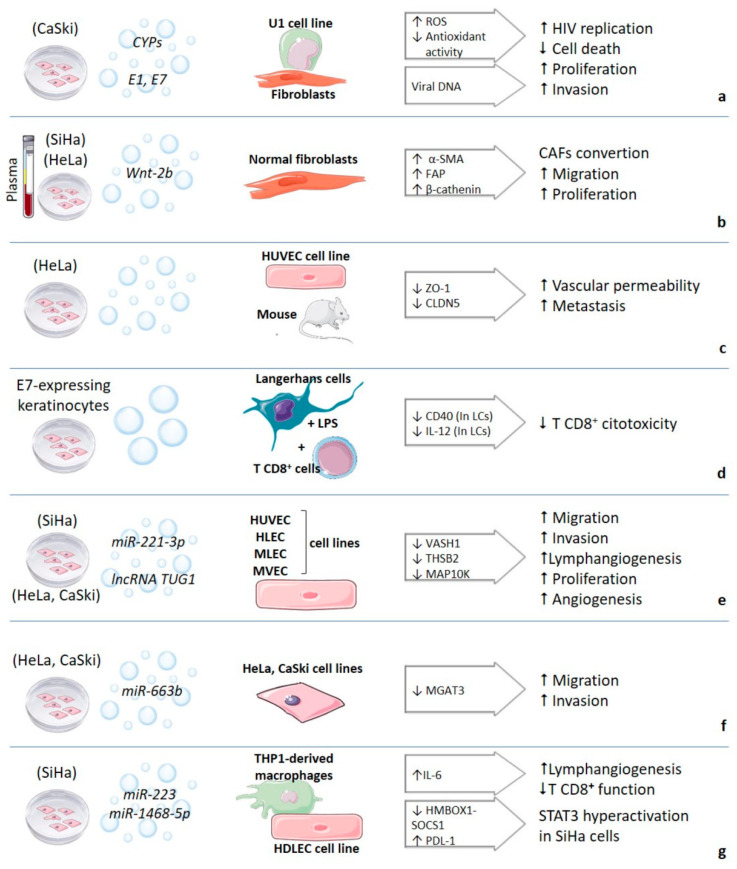
Functional effect of EVs uptake in recipient cells. Molecules in exosomes to which the effect is attributed are shown in italics and those altered in recipient cells in the arrow. Receptor cells are shown in boldface. References are indicated in square brackets. CYPs: Cytochromes P450, ROS: Reactive Oxygen Species, HIV: Human Immunodeficiency Virus, CAFs: Cancer-Associated Fibroblasts, LCs: Langerhans Cells, LPS: Lipopolysaccharide. Small arrows indicate increase or decrease of the molecules or processes indicated. References for a: [24,42]; b: [25]; c: [26]; d: [33]; e: [55,56,57,64]; f: [58]; g: [59,60]. Some images are from smart.servier.com.

Moreover, E7 HPV16 oncoprotein has also been identified in exosomes from serum from oropharyngeal cancer patients (a type of head and neck cancer). These EVs contain E7 and other cellular proteins promoting the epithelial-mesenchymal transition and invasion [34]. These reports agree with a specific protein fingerprint in EVs revealing the physiological state of their origin cells and raise the possibility of viral oncogenes working far the cervix. The proteins in EVs and the cellular processes altered due to their internalization by different cell types are represented in Figure 1. The main effects of EVs´uptake and the receptor cells identified are depicted in Figure 2.

## 3. Genomic DNA and Human Papillomavirus DNA in Extracellular Vesicles

The current knowledge about exosomes contemplates their nucleic acid content, including mitochondrial and genomic DNA [35,36]. This DNA in EVs can be single and double-stranded [36,37] and reflects the cell status so that we could detect genomic amplificated or deleted regions. In cervical cancer or preneoplastic lesions, it is assumed that the genomic level in EVs would reflect the cell condition where EVs come from, but it was only recently proved. In 2019, Thippabhotla et al. compared the DNA of EVs derived from 3D and 2D cell cultures, and they reflected their origin cells status independently of the culture or grown conditions [38].

Over the last years, several studies had suggested the presence of cervical HPV DNA in EVs due to the detection of circulating HPV DNA in patients with cervical cancer or precancerous lesions. The sequences of circulating HPV DNA were detected as derived from cervical cells with integrated genomes and in patients without cervical lesions [39,40]. More evidence for HPV DNA content in EVs was provided by De Carolis et al. in 2018. These authors found HPV in serum-derived EVs of two patients with breast cancer and a patient with benign phyllodes tumor [41]. In 2019 enough evidence for HPV DNA in EVs was provided by different groups. De Carolis et al. considered the controversial origin of the DNA in EVs as a possible effect due to vesicles uptake, looked for the type of HPV in serum-derived vesicles and that in cervical and breast cancer tissues of those who provided the serum, and found correspondence in the viral types. They also proved the presence of HPV DNA in EVs isolated from CaSki cells by digital PCR for E1 and E7 oncogenes and their transference to HPV negative recipient cells such as breast cancer-derived fibroblasts, increasing their proliferation and invasion [42].

On the other hand, Ambrosio et al. isolated plasma-derived exosomes from a male patient with colorectal cancer (squamous cell cancer) and HPV type 16 integrated into the lesion site. They found the HPV DNA type 16 in these exosomes; then, they hypothesized that the cells in the colon acquired this DNA by exposing them to exosomes with HPV DNA. But, the HVP presence in keratinocytes-derived exosomes needs to be proved. So, they also isolated exosomes from CaSki cells and confirmed the presence of HPV DNA by PCR for L1, E1, and E6 genes. These authors also demonstrated this DNA transference to human colon cancer and regular cell lines [43].

We also evaluated the DNA content in exosomes derived from the HeLa cell line through Next-Generation Sequencing (NGS). After eliminating the human DNA, we could identify HPV DNA presence. The identified fragments corresponded to the expected integrated status in this cell line. Due to viral genomes are usually integrated into cervical cancer, but not in the low-grade lesions, exosomes obtained from cervical samples were also evaluated looking for HPV DNA, despite the expected episomal status. NGS and PCR could identify HPV DNA in HPV positive but not in those negative samples. Furthermore, keratinocytes could be identified as the possible origin of some isolated exosomes thanks to the identification of cytokeratin 10 [44]. EVs’ investigation in cervical cancer seems a promising area, but the role of this exosomal DNA in the transformation or progression to cancer and their actual impact on several kinds of infected cells need still further investigation.

## 4. RNA in Extracellular Vesicles

The RNA content (including mRNAs, miRNAs, lncRNAs, and circRNAs) in EVs and their function have also been evaluated in cervical cancer or premalignant lesions since 2013. This content seems to be selectively loaded in EVs. Therefore, the RNA relative abundance differs from that observed in their origin cells, and most analyses focus on the miRNAs content [45,46,47,48]. In this section, we summarize the main findings in this regard.

In 2013, a complete exosome workflow was proposed to evaluate the exosomal miRNAs and other RNAs using HeLa cells as a model. The primary RNAs packaged in exosomes were different from those in HeLa cells. The most abundant miRNA in exosomes from HeLa cells was hsa-mir-21. Notably, when the authors evaluated blood serum samples, hsa-mir-21 was not detected among the top ten miRNA in exosomes [46]. mir-21 and mir-146a, two miRNAs associated with migration and invasion in cervical cancer, were the first identified in EVs (exosomes) from cervicovaginal lavages of women with cervical cancer or HPV infection. Their presence was also confirmed in exosomes from HeLa cells. In addition, the authors demonstrated through a luciferase indicator assay that mir-21 from exosomes of HeLa cells was able to exert a functional effect in 293T receptor cells [49]. The miR-146a-5p and miR21-5p were also increased in plasma exosomes from cervical cancer patients. This result was consistent with the increased levels observed in serum, and miR-146a-5p was also up-regulated in tumor tissues [50].

Some experiments with cell lines have shown that HPV influences the EVs’ content. To evaluate this effect, several cell lines HPV+ or HPV- have been analyzed [45]. Cell lines with silenced [48,51] or induced [52,53] HPV E6/E7 oncogenes have also been evaluated. Silencing HPV E6/E7 oncogenes in HeLa cells, surprisingly increased miR-21-5p (considered pro-tumorigenic) levels [48]. This result highlighted the need for an in-deep analysis to identify this miRNA in exosomes, to investigate how these oncogenes’ overexpression induces up-regulation of this miRNA in cells [52] and how it can be even detected in exosomes [53].

Other authors have observed that silencing HPV E6/E7 oncogenes in HeLa cells also increases miR-377 packaged levels in MVs. These MVs inhibited endothelial cell angiogenesis by binding miR-377 to the pro-angiogenic molecule LPAR [51]. Other miRNAs such as let-7d-5p, miR-20a-5p, miR-378a-3p, miR-423-3p, miR-7-5p, miR-92a-3p are also downregulated in exosomes due to E6/E7 silencing [48]. The induced expression of E6/E7 or the comparative analysis between HPV positive and negative cell lines has evidenced other miRNAs in exosomes. miR-21-5p, let-7-5p, or mir-222, among others, have been consistently modified due to the presence of HPV or the E6/E7 oncogenes [45,48,50,52,53].

Other miRNAs identified in EVs include miR-30d-5p and let-7d-3p. These miRNAs were identified in EVs from cervical intraepithelial neoplasia (CIN I, II, III) and cervical cancer patients. In this study, the authors referred that these miRNAs might be used to discern between different degrees of the lesion [47]. Still, other profiles have been observed when cervical or vaginal lavages are used [54]. Nevertheless, in addition to serving as potential biomarkers, miRNAs packaged in EVs may also have an active effect on disease promotion thru binding target molecules in recipient cells. The exosomes from E6/E7-transduced keratinocytes or SiHa and HeLa cell lines could promote tumor progression and inhibit apoptosis or promote cell proliferation and angiogenesis [48,52,53]. Several studies have identified the presence of miR-221-3p in exosomes from cell lines and plasma samples from cervical cancer patients. The binding capacity of miR-221-3p to two molecular targets VASH1 and THSB2, which function as angiogenic inhibitors, has been demonstrated [55,56]. The binding of miR-221-3p to MAPK10 has also been reported to enhance migration, invasion, and angiogenic abilities in cervical cancer cells [57].

Another exosomal miRNA associated with cancer-promoting processes is miR-663b. After TGF-β exposure of HeLa and CaSki cells, there was a selective enrichment of this miRNA in the exosomes released by the cells. The authors associate this effect with increased migration and invasion observed after capturing their exosomes. They also propose that the miR-663b mechanism targets the glycosyltransferase MGAT3, a molecule involved in regulating cell adhesion in receptor cells [58].

In addition, an essential role of vesicular miRNAs from cervical cancer in immune regulation has been established [21]. For example, the exosomal miR-223 from SiHa cells was able to induce IL-6 secretion in THP-1-derived macrophages. This IL-6 promoted STAT3 activation in SiHa cells and consequently a positive regulation of miR-223. This positive feedback through STAT3 hyperactivation could be critical for cell cycle progression and neoplastic transformation [59]. Another miRNA, the exosomal miR-1468-5p, can suppress HMBOX1-SOCS1 expression and activate JAK2/STAT3 signaling in lymphocytes promoting immune escape of cancer cells and tumor progression [60].

Despite the current knowledge, clarifying the action mechanisms and employing some miRNAs as cervical cancer biomarkers require further analysis to elucidate the discrepancies in small RNAs profiles in exosomes from different cell types and samples. Recently, Thippabhotla et al. evaluated the small RNAs in exosomes derived from cells cultured in 3D, 2D, or plasma from patients with cervical cancer. They found that the observed profile in exosomes from 3D cultures was highly similar to that in exosomes from plasma. But, those small RNAs kept in exosomes from 2D culture did not evidence the specific expected sorting, and it was comparable to the profile of small RNAs in their cells [38].

The lncRNAs described in EVs include lincRNA-p21, CCND1-ncRNA, HOTAIR, TUG1, and GAS5. These lncRNAs were selectively contained in exosomes when damage in DNA was induced with bleomycin [61]. Other lncRNAs, like HOTAIR, MALAT, and MEG3, are differentially loaded in exosomes according to the HPV infection status or the degree of the lesion [62]. lncRNA DLX6-AS1 seems to increase in serum exosomes of cervical cancer patients, but not in cervical intraepithelial neoplasia patients, and its presence is associated with a poor prognosis [63]. The effect of exosomal lncRNAs in receptor cells or the mechanisms employed has also been evaluated. For example, the lncRNA TUG1, identified in exosomes derived from HeLa and CaSki cell lines, promotes proliferation and angiogenesis-associated events in HUVEC cells upon exosome uptake [64].

Additionally, the exosomes secreted by HeLa cells could promote cell proliferation, apoptosis, and drug resistance in recipient cells by promoting the expression of TUFT1, a protein associated with poor prognostic in several cancers. This effect is facilitated by the lncRNA HNF1A-AS1 [65]. The lncRNA LINC01305 was enriched in exosomes from C-33 A cells (HPV negative) and promoted cervical cancer progression in vivo and in vitro. When C-33 A cells were treated with exosomes containing overexpressed LINC01305, the authors observed an increase in p65 and STAT3 proteins. In vivo experiments showed that there was increased tumor growth in mice treated with these exosomes. In addition, the authors reported that co-culturing LINC01305-enriched exosomes with C-33 A cells generated an increase in β-catenin, TCF7, and CCND2 protein expression, suggesting that Wnt signaling may be associated with the LINC01305 effect [66].

Other ncRNAs types like the circRNA-PVT1 have been reported in plasma and urine exosomes of patients with cervical cancer. These circRNAs transported in exosomes could promote processes associated with migration and invasion by favoring the expression of molecules related to epithelial-mesenchymal transition such as Vimentin, N-cadherin, and SNAIL [67]. However, more evidence is still necessary to confirm all these results.

The first analysis about mRNA in EVs showed a group enriched in exosomes from the HeLa cell line. These include SUSD2, BRWD3, SENP6, FAM59B, TUBBA4, QRFPR, MDK, MTRNR2L2, CWC25, DUSP13. But, this analysis mainly shows that mRNA packaging into different EVs’ types is a selective process [46]. In addition, the presence of elevated PI3k, Akt, and mTOR mRNAs was identified in exosomes isolated from vaginal secretions of women with cervical cancer. The authors suggest that this signaling pathway might promote cell proliferation and survival, which need to be evaluated in recipient cells [68].

On the other hand, Iuliano et al. in 2018 reported that HPV E6/E7 oncoproteins could affect mRNAs packaging of inflammatory cytokines in EVs released from HKF cells transduced with E6/E7 of different HPV. They evaluated mRNAs levels of various cytokines and chemokines in EVs released from HFK cells transduced with E6/E7 HPV16 or HPV38 and compared them with mRNAs levels in control HFK EVs. The differential found profiles indicate that the viral type and the presence of viral oncogenes could influence the exosomal mRNAs sorting [69]. The most recent evidence shown that the Wnt7b mRNA levels packaged in EVs, released from HeLa, SW756, SiHa, and CaSki cell lines, can be downregulated after silencing HPV 16/18. The Wntb7 mRNA contained in exosomes of these cell lines was uptaken by HUVEC cells, which promoted proliferative and pro-angiogenic events through β-catenin signaling. The levels of Wnt7b mRNA were elevated in EVs obtained from the serum of cervical cancer patients. The authors proposed that the protein synthesis may follow the uptake of EVs with Wnt7b mRNA in the receptor cell [70]. Alike the miRNAs, mRNAs cargo in exosomes seems to be also influenced by HPV status.

mRNA encoding for HPV16 E6/E7 oncogenes was located into exosomes when HFK were transduced with HPV16 E6/E7 and exosomes released from the SiHa cell line. The origin of these messengers are the cells where exosomes came from, but their likely uptake by other cells and their effect remains unknown [52].

## 5. Conclusions

In cervical cancer, the main content of EVs has been analyzed. Like in other illnesses, it has been demonstrated that EVs could contain proteins and nucleic acids. Even not all viral proteins have been localized in EVs; it has been possible to identify the E6 oncoprotein in EVs of CaSki cells, a cell line of cervical cancer. In addition, it has also been determined that EVs can contain genomic and viral DNA. The RNAs in EVs include those from the cellular origin and viral mRNAs coding for E6/E7 oncogenes. Other molecules such as purine metabolites, amino acids, fatty acids, saccharides, and several other metabolites have been identified in exosomes derived from different cancers, mainly through mass spectrometry coupled to a previous separation method. The exosomes analyzed include those derived from head and neck squamous cell cancer (HNSCC), which is also related to HPV infection. But up to date, there are no reports about these molecules in cervical cancer [71,72].

EVs’characterization in cervical cancer opens broader prospects for its treatment, prevention, and prognosis. The identification of molecules contained in EVs would allow their use as markers of infection, viral integration, or disease progression. However, given the complex mixture of circulating EVs, the discrimination of their origin is very complicated. Hence, using EVs or their cargo molecules as biomarkers is still far from being their primary application. Descriptively, cervical cancer-derived EVs have been revealed as influencing hallmarks events of cancer such as angiogenesis, migration, and invasion, and probably other cell processes not related to this malignancy yet. Until now, cervical cancer therapy focused on eliminating transformed cells, but its recurrence and persistence may lead to patients’ death. Hence, to some extent, cervical cancer relapse could be due to alterations induced by EVs in the tumor milieu or far away places via blood transportation.

The comprehensive EVs´ characterization and awareness of EVs´ functions will allow their application to lead to new methods to eliminate altered cells. However, manipulating EVs content or EVs design by microparticles engineering loaded with appropriate molecules requires further EVs characterization, standardizing internalization methods, confirming receptor molecules of uptake, and evaluating cell effects and scope in various cell types.

## Figures and Tables

**Table 1:** Biomolecules identified in extracellular vesicles of cervical cancer and other types of HPV+ cancer.

**Proteins in EVs from CC and Other HPV+ Cancer**			
**EVs Type/Source**	**HPV**	**Cargo**	**Reference**
Exo HeLa	HPV18	Survivin	Khan et al., 2011
MVs HeLa	HPV18	XIAP, c-IAP1, Livin, Survivin	Honegger et al., 2013
Exo Serum from patients with oropharyngeal cancer	HPV16	MUC16, SIRPA, E7 *	Kannan et al., 2017
Exo UM-SCC-104			
Exo UM-SCC-2, UM-SCC-47, UPCI: SCC-90	HPV16	p16, Survivin, Rb, Cyclin D1, p53, SHP-2, TGF-β, FasL, OX40, OX40L, E6 *, E7 *	Ludwig et al., 2018
EVs CaSki	HPV16	CYP1A1, CYP1B1, CYP2A6, E6 *	Ranjit et al., 2020
Exo HeLa	HPV18	CHMP4B, STX-7, RPL28, and others	Lin et al., 2020
Exo SiHa	HPV16	MMP14, MMP15	
Exo Serum from patients with CC	Unknown	**Wnt-2b**	Liang et al., 2021
Exo SiHa	HPV16		
Exo HeLa	HPV18		
**RNAs in EVs from CC**			
**EVs Type/Source**	**HPV**	**Cargo**	**Reference**
microRNAs			
Exo HeLa vs HeLa cells	HPV18	**miR-21, miR-3160-1, miR-4739, miR-31, miR-23a, miR-24-2, miR-1273a, mir-30a, mir-451a, mir-1273g**	Schageman et al., 2013
Exo Cervicovaginal lavages from CC patients and HPV+ without CC patients	HPV18	**miR-21, miR-146a**	Liu et al., 2014
Exo HeLa			
Exo E6-E7-silenced HeLa	HPV18	**miR-21-5p**	Honegger et al., 2015
		*let-7d-5p, miR-20a-5p, miR-378a-3p, miR-423-3p, miR-7-5p, miR-92a-3p*	
Exo E6-E7-silenced SiHa	HPV16	**miR-21-5**	
		*miR-20a-5p, miR-378a-3p, miR-423-3p, miR-7-5p, miR-92a-3p*	
Exo HFK transduced with E6/E7 of HPV16	HPV16	miR-24, miR-191, miR-200c, miR-212, miR-320, miR-342-3p, miR-483-5p, **miR-222**	Chiantore et al., 2016
(K16) or HPV38 (K38)	HPV38	miR-92a, miR-320, miR-323-3p, miR-494	
Exo HFK transduced with E6/E7 of HPV16 vs. HFK-E6/E7 cells	HPV16	**miR-222-3p** *miR-16-5p, miR-200b-3p, miR-320a, miR-378a-3p*	Harden & Munger, 2017
Exo HFK transduced with E6/E7 of HPV16 vs. Exo HFK		**miR-16-5p, miR-18a-5p, miR-19a-3p, miR-25-3p, miR-93-5p, miR-103a-3p, miR-106b-5p, miR-107, miR-130a-3p, miR-148b-3p, miR-155-5p, miR-182-5p, miR-195-5p, miR-218-5p, miR-222-3p, miR-335-5p, miR-375, miR-625-3p, miR-652-3p** *let-7i-5p, miR-21-5p, miR-22-3p, miR-34a-5p, miR-151a-3p, miR-192-5p, miR-200b-3p, miR-205-5p, miR-221-3p, miR-320a, miR-376c-3p, miR-378a-3p*	
Exo Plasma from CC patients	Unknown	miR-146a-5p, miR-151a-3, miR-2110	Ma et al., 2019
Exo Plasma from CC and CIN II, III patients	Unknown	*let-7d-3p and miR-30d-5p*	Zheng et al., 2019
Exo Serum from CC patients	Unknown	**miR-221-3p**	Zhou et al., 2019; Wu et al., 2019; Zhang L. et al., 2019
Exo SiHa	HPV16		
Exo Cervicovaginal-fluid from CC patients	HPV16	**miR-6746-3p, miR-4667-5p, miR-6775-5p, miR-802, miR-4477a, miR-6815-5p, miR-3190-3p, miR-5095, miR-6865-5p, miR-6786-3p, miR-4669, miR-6829-5p, miR-3619-3p, miR-4778-5p, miR-6867-5p**	Wu et al., 2020
		*miR-363-5p, miR-621, miR-6810-3p, miR-6728-3p, miR-3178, miR-211-5p, miR-3927-5p, miR-3145-5p, miR-4322, miR-548t-5p, miR-4436a, miR-5700, miR-645, miR-299-5p, miR-1911-3p*	
MVs E6-E7-interfering HeLa	HPV18	**miR-377**	Zhang Y. et al., 2020
Exo SiHa	HPV16	**miR-223**	Zhang J. et al., 2020
Exo HPV+: SiHa, CaSki, C4I, SW756, SCC-154, HeLa, SCC-090, SCC-047	HPV16	**miR-1306-5p, miR-193b-5p, miR-92b-3p, miR-92b-5p, miR-365b-3p, miR-125a-5p, miR-let-7b-5p**	Tong et al., 2020
vs Exo HPV-: UPCI-068, UPCI-017, SCC-4, SCC-1, HT-3, C-33 A	HPV18		
Exo Serum from CC patients	Unknown	**miR-1468-5p**	Zhou et al., 2021
Exo SiHa	HPV16		
Exo HeLa	HPV18	**miR-663b**	You et al., 2021
Exo CaSki	HPV16		
lncRNAs			
Exo HeLa cells vs HeLa cells	HPV18	**HOTAIR, lincRNA-p21, GAS5, TUG1, CCND1-ncRNA**	Gezer et al., 2014
Exo Cervicovaginal lavages from CC patients and HPV+ without CC patients	Unknown	**HOTAIR, MALAT1**	Zhang et al., 2016
		*MEG3*	
Exo HeLa (DDP-resistant)	HPV18	**HNF1A-AS1**	Luo et al., 2019
Exo HeLa	HPV18	**TUG1**	Lei & Mou, 2020
Exo CaSki	HPV16		
Exo Serum from CC and CIN patients	Unknown	**lncRNA DLX6-AS1**	Ding et al., 2021
Exo C-33 A	Negative	**LINC01305**	Huang et al., 2021
CircRNA			
Exo Plasma and urine from CC patients	Unknown	**circ_PVT1**	Wang et al., 2020
mRNAs			
Exo HeLa vs HeLa cells	HPV18	**SUSD2, BRWD3, SENP6, FAM59B, TUBBA4, QRFPR, MDK, MTRNR2L2, CWC25, DUSP13**	Schageman et al., 2013
Exo HFK transduced with E6/E7 of HPV16 (K16)	HPV16	E6/E7 *	Chiantore et al., 2016
Exo SiHa			
EVs HFK transduced with E6/E7 of HPV16 (K16) or HPV38 (K38) vs. EVs HFK	HPV16	**CXCL10**	Iuliano et al., 2018
		*CCL27, CCL20, CXCL3, CXCL1, IL-1α, IL-1β, angiogenin*	
	HPV38	**CCL2, TNFα**	
		*CCL27, CXCL3*	
Exo Vaginal secretions from CC patients	Unknown	**PI3k, Akt, mTOR**	Zhang W. et al., 2019
Exo Serum from CC patients Exo HeLa and SW756	Unknown	**Wnt7b**	Qiu et al., 2020
	HPV18		
Exo SiHa and CaSki	HPV16		
**DNA in EVs from CC and other HPV+ cancer**			
**EVs Type/Source**	**HPV**	**Cargo**	**Reference**
EVs Serum from patients with breast cancer	HPV16	E6 *	De Carolis et al., 2018
EVs Serum from patients with breast cancer	HPV53	L1 *	De Carolis et al., 2019
EVs CaSki	HPV16	E1 *, E7 *	
Exo Plasma from patients with colorectal cancer	HPV16	L1 *	Ambrosio et al., 2019
Exo CaSki		E1 *, E6 *, L1 *	
Exo HeLa	HPV18	E1 *, E6 *, E7 *, L1 *	Mata-Rocha et al., 2020
Exo from CC patients	Unknown	E6 *, E7 *	

* Viral molecules, Boldface type: increased levels, Italics: decreased levels, MVs: Microvesicles, Exo: Exosomes, EVs: Extracellular Vesicles, HFK: Human Foreskin Keratinocytes, CC: Cervical Cancer, CIN: Cervical Intraepithelial Neoplasia, DDP: Cisplatinum.
